# Year-independent prediction of rice grain protein content using machine learning with agronomy-aligned multi-year field data

**DOI:** 10.3389/fpls.2026.1818096

**Published:** 2026-06-01

**Authors:** Hyun-Jin Jung, Yun-Ho Lee, ChungGen Lee, Seong-Woo Cho, Tae-Young Hwang

**Affiliations:** 1 Winter Crop Research Division, National Institute of Crop and Food Science, Rural Development Administration, Wanju, Republic of Korea; 2 Crop Production and Physiology Division, National Institute of Crop and Food Science, Rural Development Administration, Wanju, Republic of Korea; 3 Central-Northern Region Crop Research Center, National Institute of Crop and Food Science, Rural Development Administration, Suwon, Republic of Korea; 4 Department of Smart Agro-Industry, Gyeongsang National University, Jinju, Republic of Korea; 5 Department of Crop Science, Chungbuk National University, Cheongju, Republic of Korea

**Keywords:** anomaly-based analysis, machine learning, multi-year field experiments, replicate aggregation, rice grain protein content, year-independent validation

## Abstract

Rice grain protein content exhibits substantial inter-annual variability due to interactions between management practices and environmental conditions, making robust prediction under realistic field conditions challenging. This study evaluated the year-independent predictability of rice grain protein content using multi-year field data and machine learning approaches, with a focus on data structuring and validation strategies. Models were trained using both plot-level and replicate-mean datasets, and evaluated using leave-one-year-out cross-validation to simulate prediction under unseen growing seasons. Replicate aggregation consistently improved predictive robustness by reducing plot-level variability, while year-independent validation provided a more conservative and realistic assessment of model generalization. Despite this stringent framework, predictive performance remained relatively stable, and residual diagnostics indicated no clear systematic bias across protein levels or nitrogen application rates. Anomaly-based analysis further suggested that deviations from year–variety baselines could be predicted with meaningful accuracy, indicating that relative variations beyond dominant temporal effects were effectively captured. These results suggest that aligning data structuring and validation strategies with agronomic experimental design may be important for reliable year-independent prediction. The proposed framework may provide a transferable approach for integrating machine learning with agronomic knowledge to support crop quality prediction and adaptive nitrogen management under variable field conditions.

## 1 Introduction

Rice grain protein content is a key determinant of both the nutritional quality and end-use properties of rice and influences eating quality, texture, processing suitability, and market value (Chen et al., 2022; Lou et al., 2023; Jukanti et al., 2025). In many rice-producing regions, consumer preferences and quality-based pricing systems increasingly emphasize grain quality attributes beyond yield, reflecting the growing demand for rice products with consistent eating and processing characteristics (Cuevas et al., 2016; Fang et al., 2024). Consequently, understanding and predicting the variability in rice grain protein content under field conditions has become an important objective in agronomic research, crop quality assessment, and precision rice production (Kaneko et al., 2016; Lee et al., 2020; Liang et al., 2021).

Rice grain protein content varies substantially in response to management practices and environmental conditions (Zhu et al., 2018). Nitrogen fertilization is widely recognized as a dominant driver of protein accumulation. In contrast, temperature, solar radiation, and other climatic factors during the grain-filling period further modulate nitrogen uptake, remobilization, and assimilation into grain proteins (Tang et al., 2019; Wang et al., 2021; Shi et al., 2022). During grain filling, a large proportion of grain nitrogen is supplied through remobilization from vegetative tissues, a process that is highly sensitive to thermal and radiation conditions (Yoneyama et al., 2016). Environmental stresses, such as excessive rainfall or heat events, can disrupt nitrogen translocation dynamics and protein synthesis, leading to pronounced variability in the final grain protein content under open-field conditions (Yan et al., 2021; Fernando et al., 2025). As a result, inter-annual climate variability can induce substantial year-to-year fluctuations in protein content, even under similar management regimes.

Conventional statistical approaches have provided valuable insights into the effects of nitrogen management and environmental drivers on rice protein content, particularly in controlled field experiments. However, many such approaches rely on linear assumptions and are commonly evaluated using within-year analyses or randomly partitioned datasets that do not explicitly account for the temporal structure (Oh et al., 2019; Yang et al., 2024). These validation strategies may overestimate the predictive performance and limit their applicability to future growing seasons. In practical agricultural settings, predictive models are rarely applied retrospectively within the same year. However, they are expected to inform management decisions under unseen climatic conditions, underscoring the importance of year-independent model evaluation (Choi et al., 2025).

Recent advances in machine learning offer flexible tools for modeling nonlinear relationships in agricultural systems and have been increasingly applied to predict crop yield, biomass, and other biophysical traits using high-dimensional environmental and management data (De Clercq and Mahdi, 2024; Sah et al., 2024). However, compared with yield prediction, the applications of machine learning to grain quality traits, including protein content, remain limited. Moreover, many existing studies have focused primarily on maximizing predictive accuracy within a given dataset, with less emphasis on robustness, generalization across years, and sensitivity to experimental noise (Bashir and Sharma, 2022; Zhang et al., 2025). This limitation is particularly critical for multi-year field experiments, where environmental heterogeneity and year-specific anomalies can strongly influence model behavior.

Agronomic field experiments are typically designed with plot-level replication to quantify treatment effects and reduce the uncertainty arising from field heterogeneity (Yan, 2021; Pagliari et al., 2022). However, in many machine learning-based studies, replicate observations are treated as independent samples or implicitly ignored, potentially inflating the effective sample size and amplifying plot-level noise. Aggregating replicate measurements to represent experimental units is a standard practice in agronomy; however, the implications of this practice for machine learning-based prediction and year-independent generalization have not been systematically evaluated for grain quality traits such as rice protein content (Singh et al., 2024; Merumba et al., 2025).

Another limitation of several predictive studies is their reliance on aggregate performance metrics. Although measures, such as the coefficient of determination and root mean square error, provide useful summaries of predictive accuracy, they offer limited insights into model reliability under specific management or environmental conditions (Aslam and Farhan, 2024; Islam et al., 2024; Sharma et al., 2024). Examination of the residual behavior and deviations from year-specific baselines can reveal systematic bias, sensitivity to management intensity, and failure modes under field conditions. Despite their diagnostic value, such analyses have rarely been integrated into machine learning-based studies of rice grain protein content (Guan et al., 2024; He et al., 2024; Wang et al., 2025).

The objectives of this study were as follows: First, we characterized the variability in rice grain protein content across multiple years under different nitrogen application rates, using multi-year field experimental data. Second, we evaluated the robustness and year-independent generalization of several machine-learning models by comparing raw plot-level observations with replicate-mean datasets under a leave-one-year-out (LOYO) cross-validation framework that reflects realistic deployment in unseen growing seasons. Third, we analyzed the residual behavior and protein content anomalies to assess model reliability and extend the interpretation beyond conventional accuracy metrics. Through this approach, we aimed to provide a robust, agronomy-aligned framework for predicting rice grain protein content under realistic field conditions and to support data-driven decision-making in precision rice production.

## 2 Materials and methods

### 2.1 Field experiment and data collection

Field experiments were conducted over three growing seasons from 2019 to 2021 at experimental paddy fields of the National Institute of Crop Science, Rural Development Administration, Republic of Korea. The experimental design included three transplanting dates (early: May 10; moderate: June 10; late: July 10), three nitrogen application levels 0, 9, and 18 kg N·10a^-1^ (equivalent to 0, 90, and 180 kg N·ha^-1^), and multiple rice cultivars. The experimental design aimed to obtain plot-level observations of rice grain protein content under controlled management practices while capturing inter-annual environmental variability representative of field conditions.

The experiment was arranged in a randomized complete block design with three replicates per treatment. Nitrogen application was the primary experimental factor and was applied at three levels, (no fertilization, standard fertilization, and high fertilization). Rice seedlings were manually transplanted at a spacing of 30 cm × 14 cm with three plants per hill. Standard agronomic management practices recommended by the Rural Development Administration were uniformly applied across all plots and years. All experimental plots were located within a single paddy field under uniform soil conditions. Although detailed physicochemical soil properties (e.g., pH, organic matter, and total nitrogen content) were not explicitly measured in this study, spatial variability in baseline soil fertility was minimized by the experimental design. The rice cultivars included in this study (listed in **Supplementary Table 2**) are japonica-type, inbred varieties commonly cultivated in South Korea.

Each treatment × replicate combination was applied to an area of approximately 330 m², within which 12 rice cultivars were cultivated. Each cultivar occupied a distinct sub-plot, resulting in an effective plot size of approximately 27.5 m² per cultivar (approximately 5.2 m × 5.2 m). This plot size was sufficient to minimize edge effects and ensure representative sampling within each treatment.

Transplanting time was categorized into early, moderate, and late groups based on consistent relative timing within each growing season, rather than fixed calendar dates, to represent management regimes across years. Although multiple rice cultivars were included to increase genetic and environmental variability, cultivar-specific effects were not explicitly modeled and were treated as background variability in the analysis.

Grain samples were collected independently from each plot (approximately 27.5 m²) at physiological maturity to represent the corresponding experimental unit. This sampling strategy ensured that each observation represented the plot-level response of the corresponding treatment × replicate unit. Panicles were sampled from multiple positions within each plot, primarily from the central area to minimize edge effects, and pooled to form a composite sample for each plot. Harvesting was conducted based on accumulated temperature criteria (approximately 1100-1200 °C after heading) to ensure consistent maturity across treatments.

After harvesting, rice grains were threshed and dehulled to obtain brown rice. Impurities and immature grains were removed using a 1.6 mm sieve prior to analysis. This sieve size was selected based on the official “Standard Methods of Agricultural Science and Technology Research” (RDA, 2012).

Grain protein content was measured using a near-infrared spectroscopy (NIRS) analyzer (Foss Infratec™ 1241, Sweden) with intact brown rice samples. For each sample, more than 200 g of brown rice was used, and measurements were repeated three times to ensure analytical reliability. The results were expressed as a percentage of dry weight. Identical sampling and analytical procedures were applied across all experimental years to ensure consistency and comparability.

The resulting dataset comprised raw plot-level observations across multiple years, nitrogen application rates, and replicate experimental units. These raw data captured both within-treatment variability arising from plot-scale heterogeneity and broader inter-annual environmental effects. To align the machine learning inputs with the experimental unit structure commonly used in agronomic field trials, replicate-mean values were additionally derived by averaging observations from the three replicates within each year and treatment combination. This parallel data structure enabled a direct comparison between raw plot-level data and replicate-mean data in the subsequent modeling and validation steps, aimed at assessing prediction robustness and year-independent generalization.

The final dataset consisted of 972 observations after data preprocessing. The dataset included multiple experimental years (2019–2021), nitrogen application levels 0, 9, and 18 kg N·10a⁻¹ (equivalent to 0, 90, and 180 kg N·ha^-1^), transplanting-time groups (early, moderate, and late), and cultivars. Although the design ensured representation across all factors, the dataset was not fully balanced due to practical field constraints. Nevertheless, each treatment combination was supported by replicate observations, enabling robust statistical analysis while reflecting realistic field variability.

### 2.2 Climatic data and processing

Climatic data were collected to characterize the environmental conditions during the grain-filling period, which play a critical role in rice grain protein accumulation. The heading date for each plot was determined based on field observations during the growing season. Heading was defined as the stage when approximately 50% of panicles had emerged. This information was recorded for each experimental unit and used to align climatic variables relative to crop developmental stages.

Daily meteorological variables, including air temperature, sunshine duration, and solar radiation, were obtained from the Jeonju Regional Office of the Korea Meteorological Administration, which provides official weather observations for the experimental region.

To align climatic conditions with crop developmental stages, all climatic variables were aggregated relative to the heading date, which was treated as day 0. Two non-overlapping post-heading periods were considered to represent the grain-filling stages: early (0–20 days after heading) and late (21–40 days after heading).

Mean air temperature for each post-heading window was calculated as:


$${\bar{\text{T}}}_{0-n}=\frac{1}{n}\sum_{d=1}^{n}T_{d},\;where\;d=1\ldots n$$

The cumulative sunshine duration (SD) and solar radiation (SR) were calculated as:


$$SD_{0-n}=\sum_{d=1}^{n}SD_{d}\;and\;SR_{0-n}=\sum_{d=1}^{n}SR_{d},\;where\;d=1\ldots n$$

Where *d* represents each day within the aggregation window.

Climatic variables for the early stage (0–20 days) were calculated directly from daily observations. Variables for the late stage (21–40 days after heading) were derived by excluding the early-stage contribution from cumulative measurements, ensuring that the two periods were statistically independent.

This non-overlapping window design was adopted to avoid multicollinearity among predictors and to enable independent evaluation of early- and late-stage climatic effects on grain protein accumulation.

All climatic variables were merged with plot-level grain protein observations based on experimental year and treatment information. No additional normalization or scaling was applied prior to model training to preserve the original physical meaning of variables. The resulting climatic dataset was used directly as the input for subsequent machine learning analyses.

### 2.3 Machine learning models and feature construction

Multiple machine learning models with varying levels of structural complexity were employed to predict rice grain protein content and evaluate the predictive robustness under year-independent conditions. Regularized linear models, including the Elastic Net and Least Absolute Shrinkage and Selection Operator (LASSO), were used to capture dominant linear relationships while accounting for multicollinearity among predictors through coefficient regularization. These models provide interpretable baselines and are well-suited for datasets characterized by correlated environmental variables and limited sample sizes.

In parallel, nonlinear models, including Random Forest (RF), Extreme Gradient Boosting (XGB), and k-nearest neighbors (KNN), were applied to represent the potentially complex and nonlinear interactions between management practices and climatic conditions during grain filling. Including models with contrasting structural assumptions enabled for the assessment of whether predictive robustness and generalization across years were sensitive to model flexibility.

The model inputs comprised management factors and developmentally aligned climatic variables, as described in the preceding sections. The nitrogen application rate was included as the primary management variable.

Climatic predictors were constructed relative to the heading date to ensure alignment with crop developmental stages during grain filling. Specifically, the mean air temperature, cumulative sunshine duration, and solar radiation were calculated for two non-overlapping post-heading windows: 0–20 days (early stage) and 21–40 days (late stage) after heading. This non-overlapping window design was adopted to avoid multicollinearity among predictors and to enable independent evaluation of early- and late-stage climatic effects.

In addition, interaction terms between nitrogen application rate and climatic variables were constructed separately for the early (0–20 days after heading) and late (21–40 days after heading) grain-filling stages, allowing independent evaluation of stage-specific nitrogen–climate interactions on grain protein accumulation. The relevance of these constructed features was further evaluated through feature importance analysis.

To quantify the independent contribution of climatic variables beyond management factors, an additional feature-group baseline comparison was conducted under the same LOYO validation framework. Four feature sets were evaluated, consisting of a management-only model including nitrogen application rate and transplanting time; a climate-only model including grain-filling climatic variables; a combined management and climate model including both management and climatic predictors without interaction terms; and a full model including management variables, climatic variables, and their interaction terms. This comparison was designed to determine whether year-independent prediction performance was primarily driven by stable management variables, climatic predictors, or their interaction effects.

Categorical variables, including variety and transplantation time, were encoded using one-hot encoding. Numerical features were standardized only for models sensitive to feature scaling (Elastic Net, LASSO, and KNN), whereas tree-based models (RF and XGB) were trained using unscaled features. No normalization across years was applied to avoid introducing information from the held-out test year into the model training under the LOYO validation framework.

Two parallel data representations were considered. Each replicate plot in the raw dataset was treated as an independent observation. In the replicate-mean dataset, observations were averaged across replicate plots within each combination of year, variety, transplantation time, and nitrogen application rate to represent the experimental units commonly used in agronomic field analyses. This structure enables direct evaluation of how replicate aggregation influences model behavior and robustness across years.

To support the relative-response analysis beyond absolute protein values, protein content anomalies were defined as deviations of the observed protein content from the mean protein content of the corresponding year–variety combination. These anomaly values were used in subsequent analyses to assess the model performance after accounting for dominant temporal and genetic effects.

Hyperparameter tuning was performed using a grid search strategy within each LOYO training fold to prevent information leakage. A predefined range of hyperparameters was evaluated for each model, and the optimal configuration was selected by minimizing the root mean square error (RMSE) using internal cross-validation within the training data. This procedure was consistently applied across all models to ensure fair comparison, and the final hyperparameter settings are summarized in **Supplementary Table 1**.

### 2.4 Model validation and evaluation

Model performance was evaluated using a LOYO cross-validation framework to assess the year-independent generalization. In each LOYO iteration, the data from all but one experimental year were used for model training, and the trained model was evaluated during the held-out year. This procedure was repeated so that each experimental year served as an independent test set, thereby reflecting realistic deployment scenarios in which predictive models were applied to unseen growing seasons characterized by distinct climatic conditions.

For comparison, an additional repeated random split validation was conducted using the same datasets and model configurations. In this approach, observations were randomly partitioned into training and test sets irrespective of year, allowing a direct assessment of how validation strategy influences the estimated predictive performance. This comparison was designed to evaluate the extent to which conventional validation strategies may overestimate model performance under multi-year field conditions.

To further distinguish the effect of year-wise separation from the effect of reduced training-set size, a size-matched random split validation was additionally performed. In this analysis, the test-set size was matched to each held-out year in the LOYO framework, while samples were randomly selected irrespective of experimental year. Thus, the size-matched random split had the same average training and test sizes as LOYO, but did not enforce year-wise separation. This comparison was designed to evaluate whether the performance decrease under LOYO validation was attributable primarily to the smaller training set or to year-wise distribution shifts.

Model training and evaluation were conducted separately for the raw plot-level and replicate-mean datasets, using identical validation procedures. To prevent information leakage, all the preprocessing steps, including feature construction and hyperparameter tuning, were restricted to the training data within each LOYO iteration. No information from the held-out test year was used during the model fitting or parameter optimization. In addition, the calendar-year variable was excluded from the model inputs to ensure that predictions for the test year were based solely on management practices and climatic conditions.

The predictive performance was quantified using the coefficient of determination (R²), root mean square error, and mean absolute error. These metrics were calculated separately for each held-out year and subsequently summarized across all LOYO folds to provide an overall assessment of the model’s robustness and generalization. This fold-wise evaluation allowed for a direct comparison of model performance across years characterized by contrasting environmental conditions.

In addition to the aggregate performance metrics, prediction residuals were retained for further diagnostic analysis. Residuals were examined to evaluate potential systematic bias across protein content levels, nitrogen application rates, experimental years, and cultivars. Residuals were further stratified by year and cultivar to assess temporal consistency and cultivar-specific bias in prediction errors.

Furthermore, residual information was used to support the anomaly-based interpretation of model behavior, enabling the assessment of relative deviations in protein content after accounting for dominant year- and variety-specific effects, as described in subsequent sections.

In addition, residual information was used to support the anomaly-based interpretation of model behavior, enabling the assessment of relative deviations in protein content after accounting for dominant year- and variety-specific effects, as described in subsequent sections.

To further assess the practical role of the anomaly-based framework, an additional evaluation was conducted by reconstructing absolute protein predictions from the predicted anomalies and year-specific baseline values, and comparing them with the direct protein prediction model.

## 3 Results

### 3.1 Variability of rice grain protein content

To establish an empirical context for subsequent predictive modeling, the variability in rice grain protein content was first examined across the full dataset. This descriptive analysis provides an overview of the magnitude and structure of variations observed under realistic field conditions.

**Figure 1:**
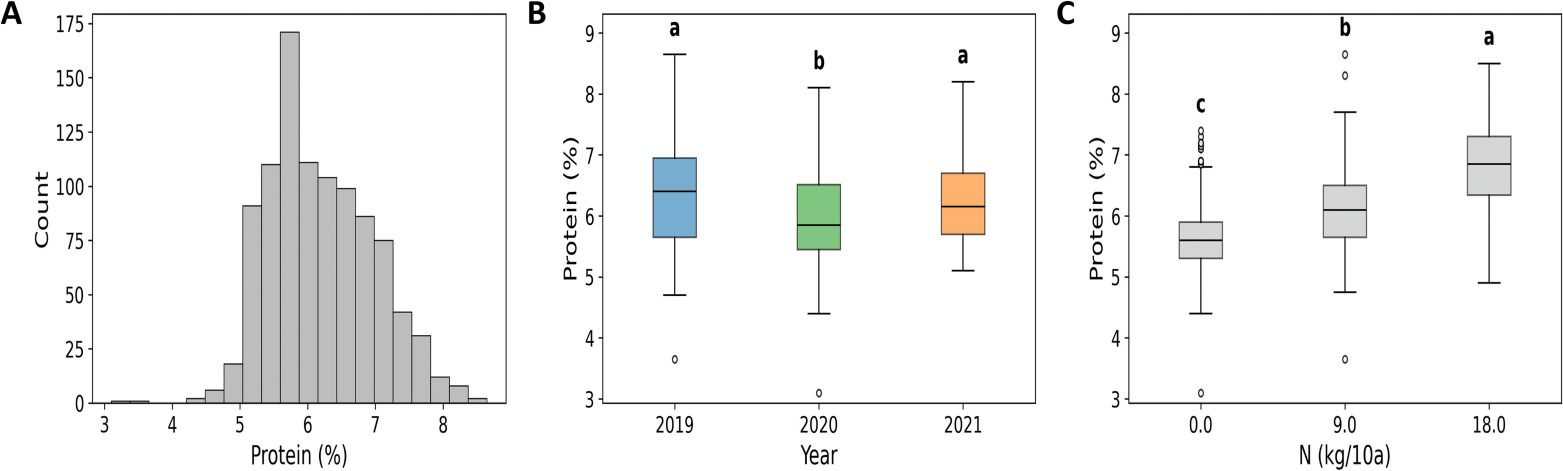
Distribution of rice grain protein content of the entire dataset and major experimental factors. **(A)** Histogram of observed protein content values. Boxplot of protein content **(B)** by experimental year and **(C)** by nitrogen application rate. Different letters above boxplots indicate significant differences among groups according to Tukey’s honestly significant difference (HSD) test at p< 0.05.

Rice grain protein content showed moderate variability across the entire dataset (**Figure 1A**). The observed values were distributed within a relatively broad but continuous range, forming a unimodal distribution centered around mid-range of protein content. This pattern indicates that protein levels were influenced by multiple interacting factors rather than being dominated by a single driver, while overall remaining within a constrained range under the given experimental conditions.

Inter-annual differences in rice grain protein content were observed (**Figure 1B**). Median protein levels varied among years, with 2020 showing relatively lower values compared to 2019 and 2021. These differences were statistically distinguishable, although the overall distribution remained partially overlapping. In addition, the spread of protein values within each year also differed, suggesting that year-specific environmental conditions contributed to variability in protein accumulation. These results indicate that inter-annual variability was present, but its magnitude was moderate relative to other sources of variation.

Protein content exhibited a clear response to nitrogen application rate (**Figure 1C**). Increasing nitrogen input was associated with a consistent increase in median protein content, and the differences among nitrogen levels were statistically well separated. Despite this systematic trend, variability within each nitrogen level remained evident, indicating that environmental and other uncontrolled factors also contributed to within-treatment variation.

Overall, rice grain protein content varied across both years and nitrogen levels, with nitrogen application exerting a stronger and more consistent influence, while inter-annual differences contributed additional but comparatively moderate variation. This combination of structured and residual variability provides a realistic basis for subsequent year-independent predictive modeling and robustness assessment. Detailed summary statistics are provided in **Supplementary Table 2**.

### 3.2 Inter-annual climatic variation during the grain-filling period

**Figure 2:**
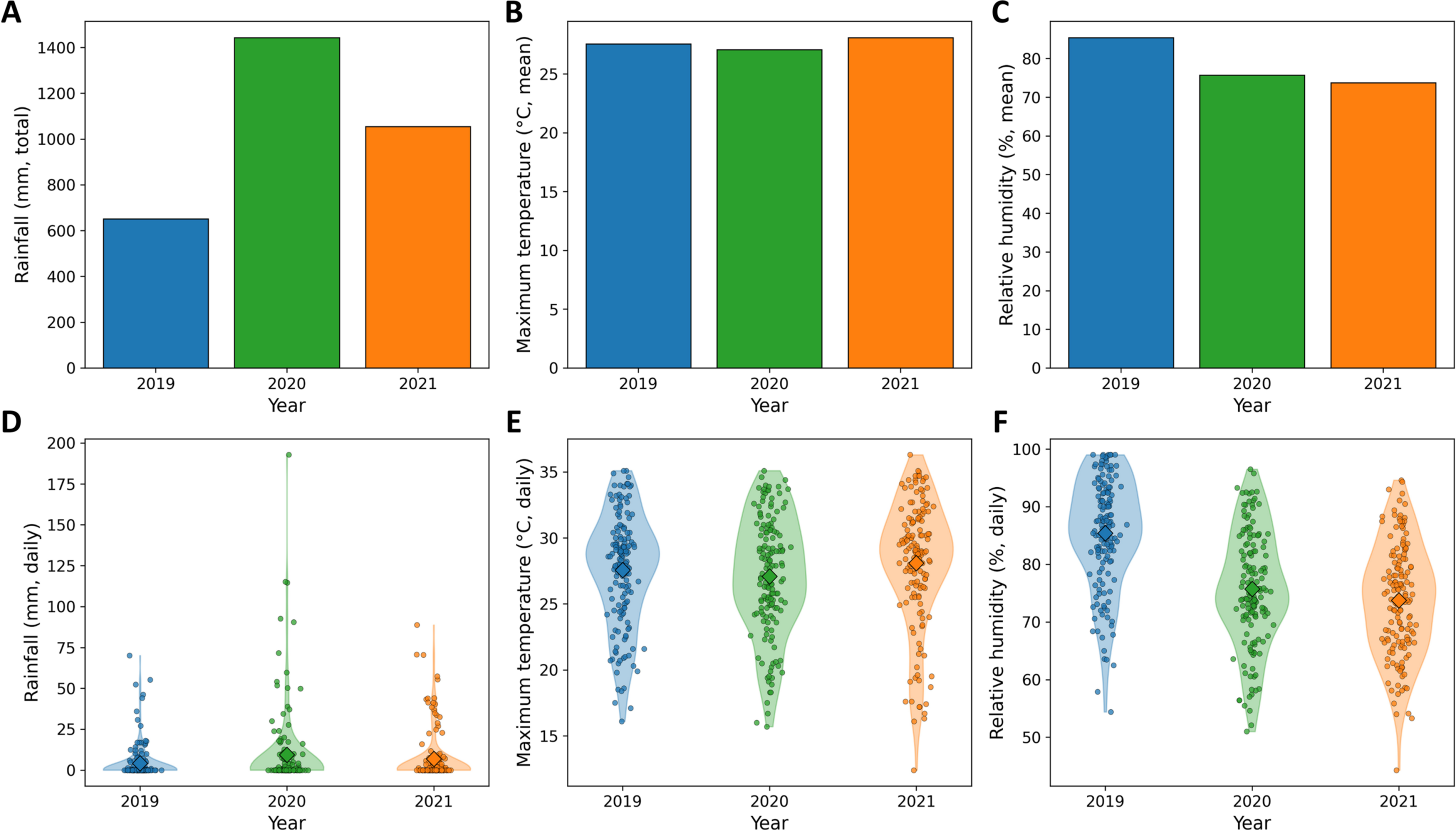
Inter-annual variation in climatic conditions during the grain-filling period. **(A–C)** summary statistics showing total rainfall, mean daily maximum temperature, and mean relative humidity for each year. **(D–F)** distribution of daily values for the same variables, illustrating within-season variability.

Inter-annual climatic conditions during the grain-filling period varied across the three experimental years (**Figure 2**). Total rainfall differed markedly, with 2020 receiving substantially higher cumulative precipitation (1443 mm) compared to 2019 (650.8 mm), while 2021 showing slightly higher values (28.1 °C) compared to 2019 (27.6 °C) and 2020 (27.1 °C). Relative humidity also varied among years, with 2019 exhibiting higher mean values (85.4%) compared to 2020 (75.7%) and 2021 (73.8%).

These results indicate that the growing seasons were characterized by differences in key climatic components, particularly rainfall and humidity, although not all variables exhibited large inter-annual contrasts. This suggests that the prediction task involved moderately varying environmental conditions rather than identical repetitions of similar climatic regimes.

Despite these climatic, the overall distributions of grain protein remained broadly overlapping across years (**Figure 1B**). This pattern indicates that inter-annual climatic variation influenced protein formation in combination with other major factors, including nitrogen application, transplanting time, and cultivar differences, rather than producing fully separated year-specific protein distributions.

### 3.3 Comparison among random split, size-matched random split, and LOYO validation

**Figure 3:**
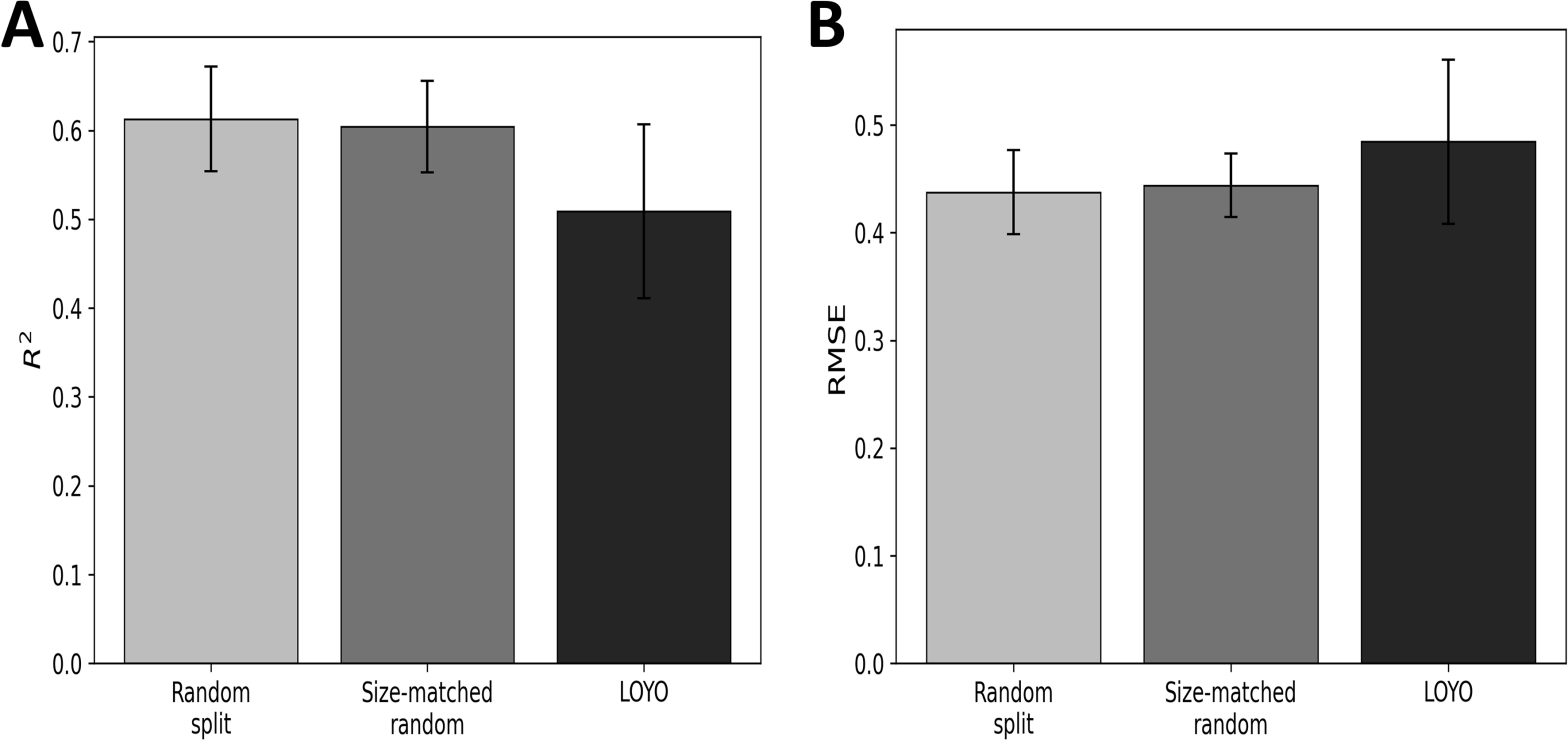
Comparison of validation strategies for the LASSO model using the replicate-mean dataset. **(A)** coefficient of determination (R2) and **(B)** root mean square error (RMSE) under repeated random split, size-matched random split, and leave-one-year-out (LOYO) validation. Error bars indicate standard deviations across validation repeats or folds.

To evaluate the impact of validation strategy, predictive performance was compared between repeated random split validation, size-matched random split validation, and leave-one-year-out (LOYO) validation (**Table 1**; **Figure 3**). Across models, random split validation produced the highest predictive performance, whereas LOYO validation resulted in more conservative estimates.

**Table 1:** Comparison of predictive performance under different validation strategies for the replicate-mean dataset using protein as the target variable.

Model	Validation	R2	RMSE	MAE
ElasticNet	Random split	0.600	0.445	0.346
	Size-matched random split	0.594	0.450	0.348
	LOYO	0.504	0.487	0.373
KNN	Random split	0.608	0.440	0.340
	Size-matched random split	0.586	0.454	0.350
	LOYO	0.376	0.547	0.426
LASSO	Random split	0.613	0.438	0.341
	Size-matched random split	0.605	0.444	0.344
	LOYO	0.509	0.485	0.368
RF	Random split	0.633	0.425	0.329
	Size-matched random split	0.611	0.439	0.337
	LOYO	0.230	0.606	0.466
XGB	Random split	0.657	0.411	0.324
	Size-matched random split	0.620	0.434	0.337
	LOYO	0.151	0.635	0.489

The size-matched random split analysis was conducted to examine whether the lower LOYO performance could be explained by reduced training-set. In this analysis, the test-set size was matched to each LOYO fold while samples were randomly selected irrespective of year. For the LASSO model, random split validation achieved an R^2^ of 0.613 and RMSE of 0.438, while size-matched random split achieved similar performance (R^2^ = 0.605, RMSE = 0.444). In contrast, LOYO validation resulted in a lower R^2^ of 0.509 and a higher RMSE of 0.485.

This pattern was consistently observed in linear models, where size-matched random split produced performance comparable to random split despite the reduced training-set size. However, larger discrepancies were observed in some non-linear models, indicating that both training-set size and model structure can influence performance. Overall, these results suggest that the additional performance decrease under LOYO cannot be attributed solely to reduced training-set size, but also reflects the impact of year-wise separation and distributional differences among experimental years.

The observed performance gap between random split and LOYO validation should be interpreted in the context of both validation design and environmental variability. As shown in **Figure 2**, the experimental years exhibited differences in key climatic variables during the grain-filling period, particularly in rainfall and relative humidity. Accordingly, LOYO validation provides a more stringent assessment of model generalization under varying environmental conditions, rather than under randomly mixed samples drawn from similar distributions.

### 3.4 Model robustness under year-independent validation

Building on the observed variability in rice grain protein content, model robustness was examined under a year-independent validation framework. Predictive performance was evaluated using LOYO cross-validation, with an explicit comparison between models trained on raw plot-level observations and those trained on replicate-mean datasets.

**Figure 4:**
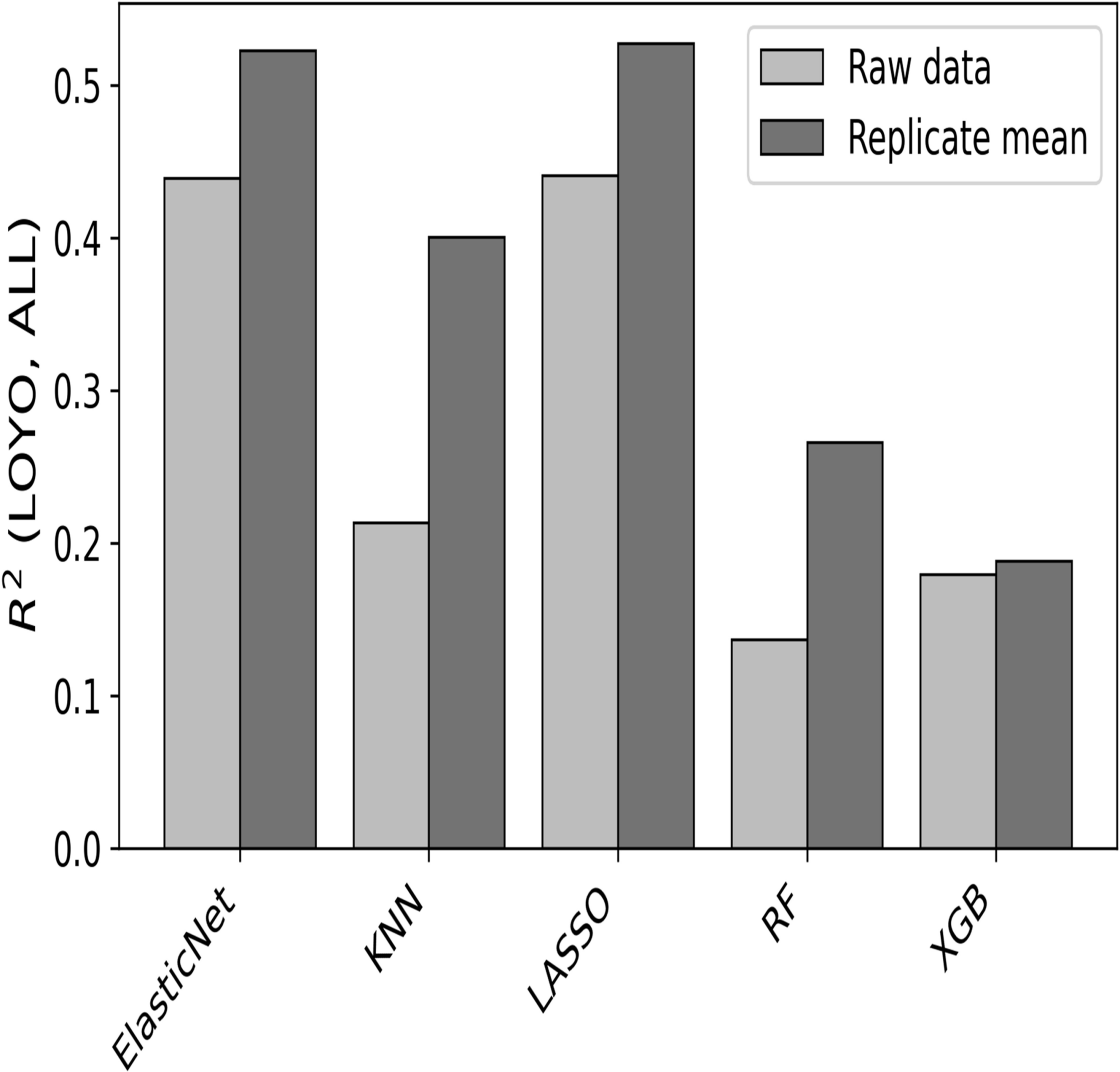
Comparison of prediction performance between raw data and replicate-mean datasets across five machine learning models evaluated using Leave-One-Year-Out (LOYO) cross-validation. LASSO, Least Absolute Shrinkage and Selection Operator; KNN, k-nearest neighbors; RF, Random Forest; XGB, Extreme Gradient Boosting.

Across most evaluated models, replicate aggregation improved predictive performance relative to raw data (**Figure 4**). This trend was observed in both the linear and nonlinear models, although the magnitude of improvement varied depending on model structure. By reducing plot-level variability associated with within-treatment noise, replicate aggregation generally enhanced model stability under year-wise partitioning.

The magnitude of performance gains differed among models, but the overall direction of the effect was largely consistent. Models trained on replicate-mean data achieved higher coefficients of determination and, in most cases, lower prediction errors under LOYO validation compare to their raw-data counterparts. These results suggest that aligning data representation with agronomic experimental units can improve robustness against inter-annual variability in field-based datasets.

The interpretation of LOYO performance should consider both validation design and environmental variability. As shown in **Figure 2**, the experimental years differed in key climatic variables, particularly precipitation and relative humidity, although the overall environmental contrast was moderate. Under these conditions, LOYO validation provides a more stringent test of model generalization compared to random partitioning.

**Figure 5:**
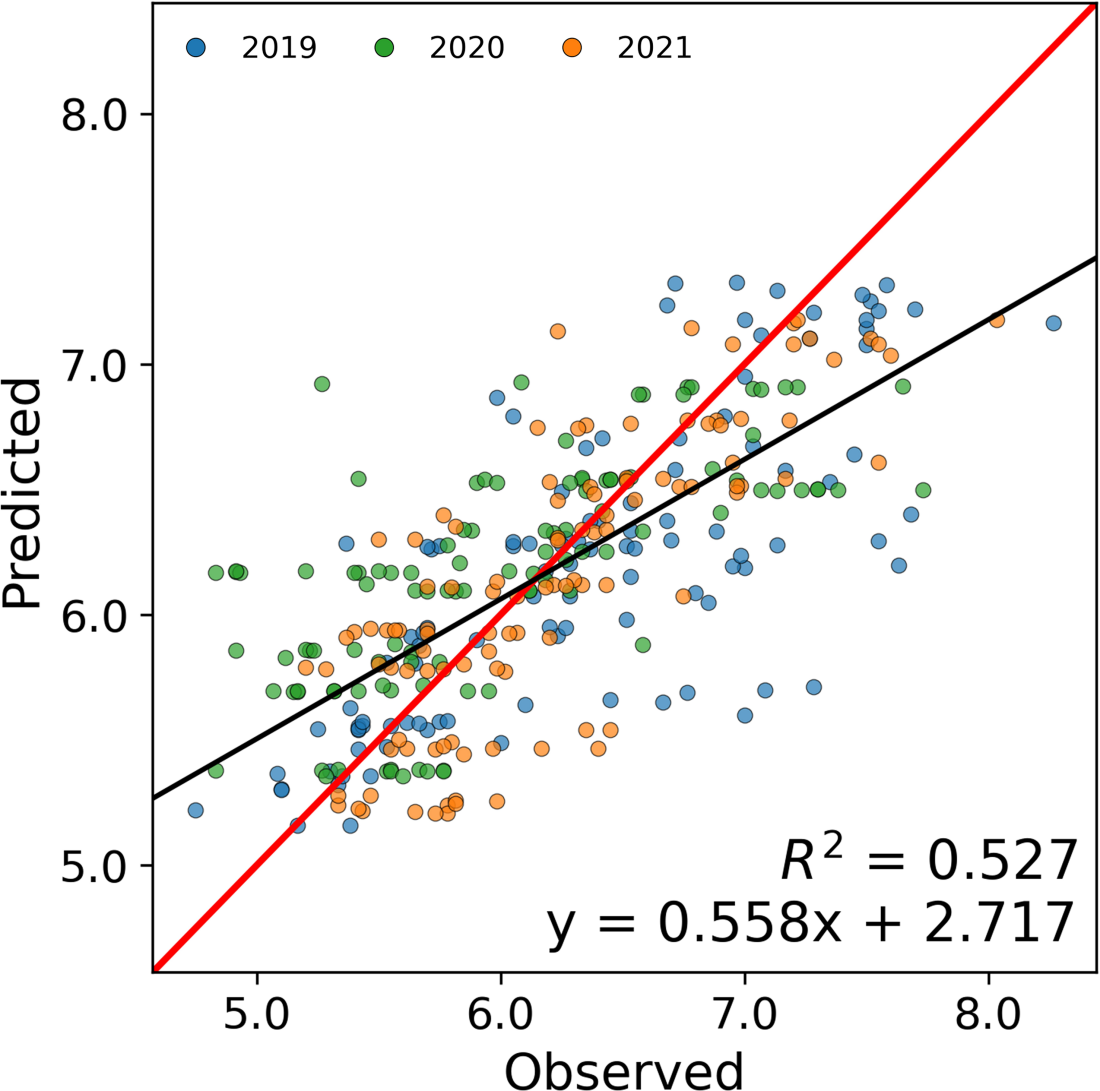
Observed versus predicted rice grain protein content obtained from the LASSO under leave-one-year-out (LOYO) cross-validation using the replicate-mean dataset. Each point represents an individual observation, colored by experimental year. The red line indicates the 1:1 relationship, and the black line represents the fitted regression between observed and predicted values.

Beyond aggregate performance metrics, differences in model behavior were evident when examining prediction dispersion across the observed range of protein content (**Figure 5**). Elastic Net and LASSO captured broad linear relationships with relatively stable dispersion across protein levels, whereas k-nearest neighbors and Random Forest exhibited greater scatter, particularly at higher protein content. This pattern suggests increased sensitivity of more flexible models to residual variability under limited training data conditions.

Extreme Gradient Boosting exhibited intermediate behavior, balancing flexibility and stability across the observed range. In addition to predictive performance, computational efficiency was also evaluated using model training time (**Supplementary Table 3**). Regularized linear models (Elastic Net and LASSO) consistently required substantially shorter training times compared to nonlinear models such as Random Forest and Extreme Gradient Boosting.

On average, the training time of linear models was considerably lower, reflecting their simpler structure and reduced computational complexity. In contrast, nonlinear models required longer training times due to their iterative optimization and ensemble-based architecture.

These results indicate that, while nonlinear models offer greater flexibility, their higher computational cost and sensitivity to data variability may limit their practicality in field-based applications. In contrast, regularized linear models provide predictive performance with substantially lower computational demand, making them more suitable for scenarios requiring rapid model updating and limited computational resources.

Overall, the relatively strong performance of regularized linear models under LOYO validation suggests that dominant linear relationships among nitrogen management, climatic conditions, and grain protein content are sufficiently informative for robust year-independent prediction when replicate aggregation is applied. Although nonlinear models may capture additional complexity, they appear more prone to fitting residual variability that does not consistently generalize across years.

Despite these differences, all models retained meaningful predictive skill under a stringent year-independent validation framework. Together, **Figure 3** and 4demonstrate the importance of data aggregation in improving robustness under realistic validation conditions. Detailed numerical performance metrics are provided in **Supplementary Table 4**.

### 3.5 Contribution of climatic variables beyond management effects

**Figure 6:**
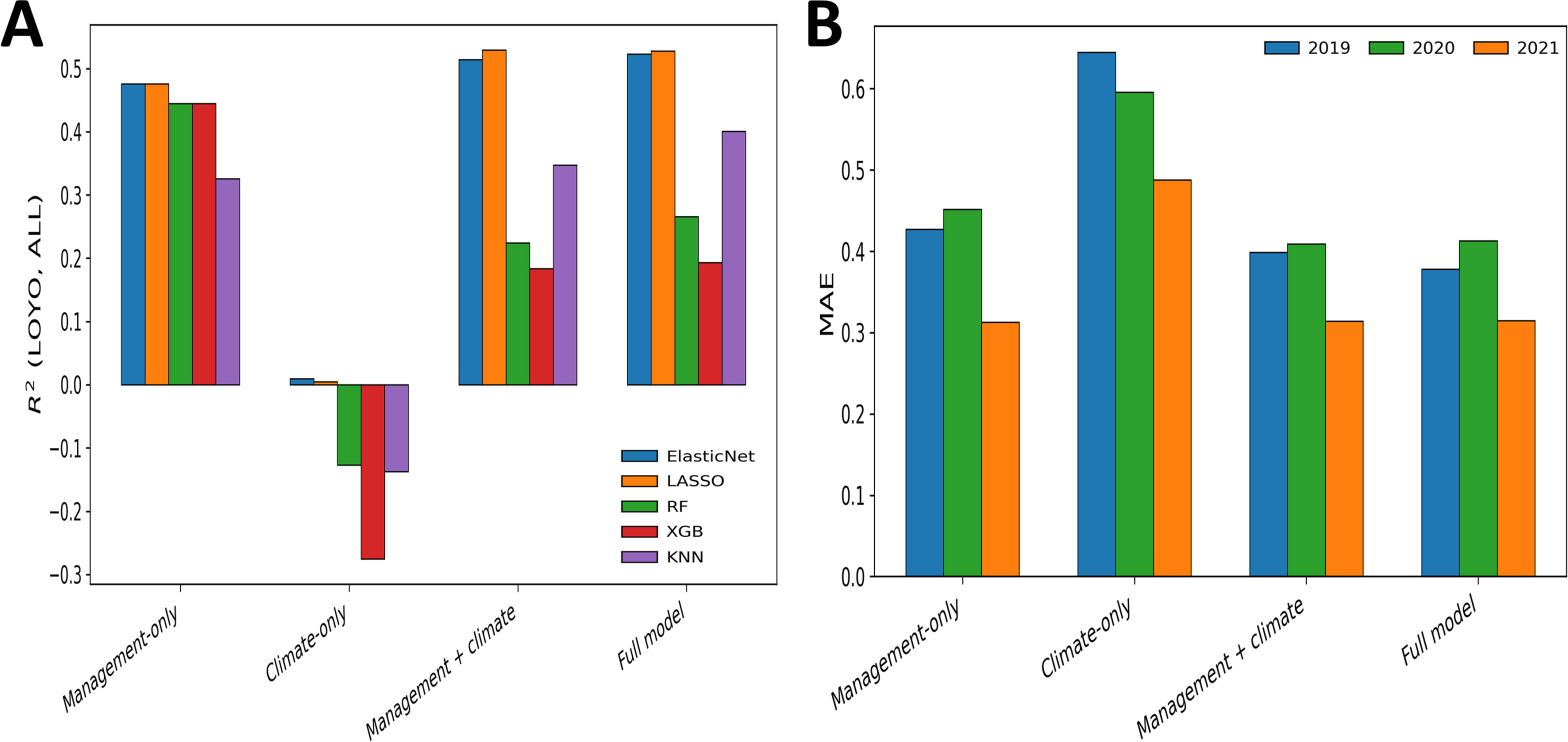
Comparison of predictive performance under the LOYO validation framework across different feature groups. **(A)** Coefficient of determination (R²) and **(B)** mean absolute error (MAE) for the LASSO model.

To determine whether climatic variables provided predictive information beyond management-related predictors, we compared four feature groups under the same LOYO validation framework (**Figure 6**).

As shown in **Figure 6A**, model using only management-related variables (nitrogen application rate and transplanting time) achieved moderate predictive performance, with R² values generally around 0.45-0.50 for linear models. The indicates that management factors alone explained a substantial portion of predictable variation in grain protein content.

In contrast, models using only climatic variables exhibited very limited predictive ability. For linear models, R^2^ values were close to zero, while several nonlinear models showed negative R^2^ values, indicating that climate-only predictors were insufficient to capture the variation in protein content under year-independent validation.

The inclusion of climatic variables alongside management factors improved predictive performance. R² values increased to approximately 0.50-0.53 for linear models, while the full model provided only marginal additional improvement. This suggests that climatic variables contribute complementary predictive information, whereas interaction effects provide relatively limited gains under the current dataset.

Consistent patterns were observed for MAE (**Figure 6B**). Model using management-only variables showed higher prediction errors, while the inclusion of climatic variables reduced MAE across most models. However, the additional improvement from interaction terms in the full model remained small.

### 3.6 Feature importance and interpretation of model predictors

**Figure 7:**
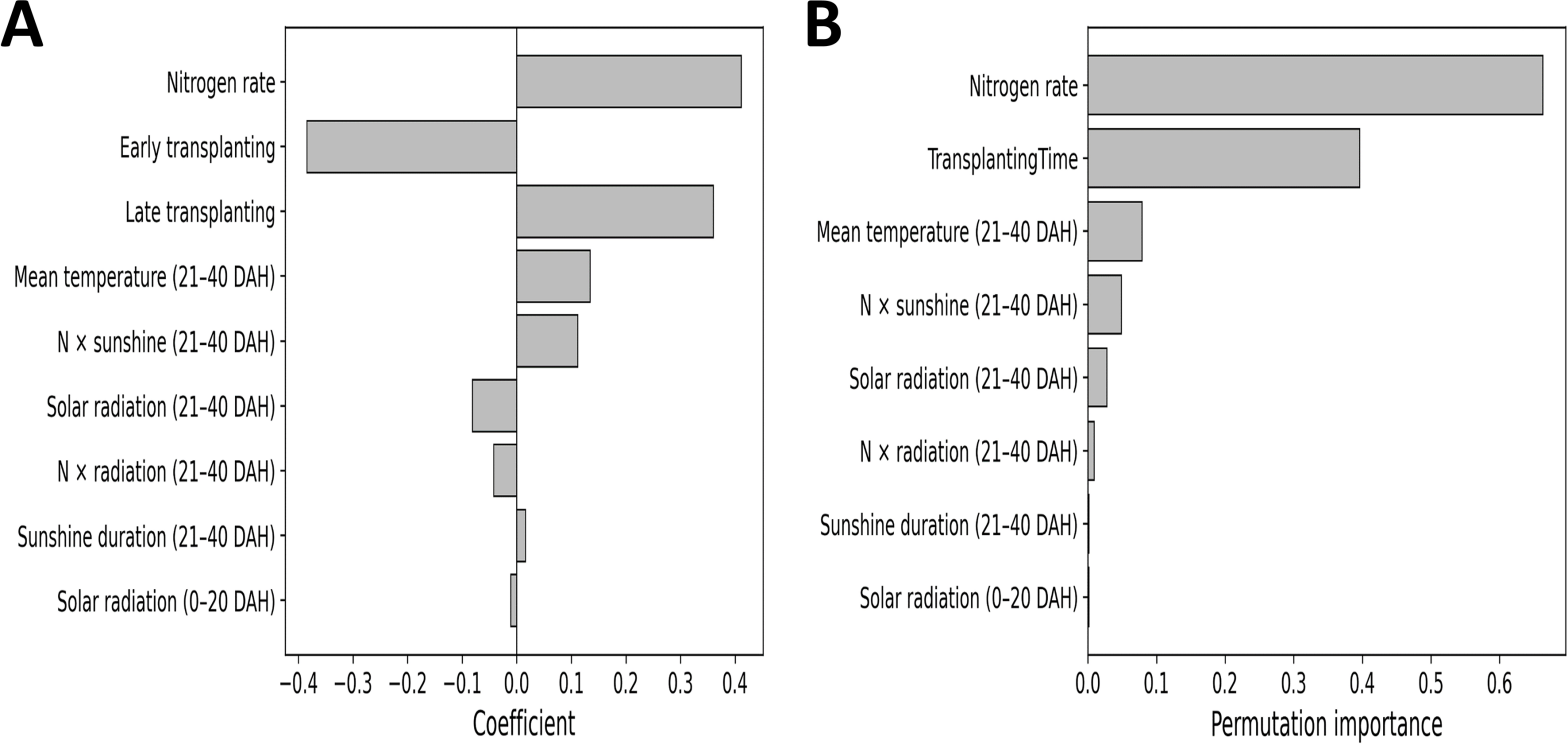
Feature-importance analysis of the LASSO trained on the replicate-mean dataset for protein prediction. **(A)** standardized model coefficients of the most influential predictors. **(B)** permutation importance of the same predictors.

To improve the interpretability of climatic predictors, feature-importance analyses were conducted for LASSO trained on the replicate-mean dataset. Both model coefficients and permutation importance were examined to identify the variables that most strongly contributed to prediction performance (**Figure 7**).

Nitrogen application rate was the most influential predictor, followed by transplanting-time category. Among the climatic variables, temperature and solar radiation during the late grain-filling stage showed relatively high importance. The feature importance results indicate that climatic variables during the late grain-filling stage (21–40 days after heading) contributed more strongly to prediction performance than those during the early stage (0–20 days after heading).

The coefficient-based interpretation further indicated that higher temperature during the late grain-filling stage (21–40 days after heading) was positively associated with protein prediction, whereas higher solar radiation during the same stage showed a negative contribution. These results suggest that the model captured biologically meaningful relationships linking grain-filling environment, nitrogen response, and final protein accumulation.

Nitrogen application rate was identified as the most influential predictor across both coefficient-based and permutation-based analyses. Transplanting time also showed a strong effect, followed by climatic variables such as temperature and radiation during the late grain-filling stage. These results suggest that the model captured biologically meaningful relationships between nitrogen availability, environmental conditions, and grain protein formation.

However, the feature-group comparison demonstrated that a substantial proportion of predictive performance was already explained by management variables alone. Therefore, the feature-importance results should be interpreted as relative contributions within the full model rather than as evidence that climatic variables independently dominated prediction performance. In this context, climatic variables should be interpreted as providing complementary predictive information rather than acting as dominant standalone predictors.

### 3.7 Residual behavior and anomaly-based interpretation

**Figure 8:**
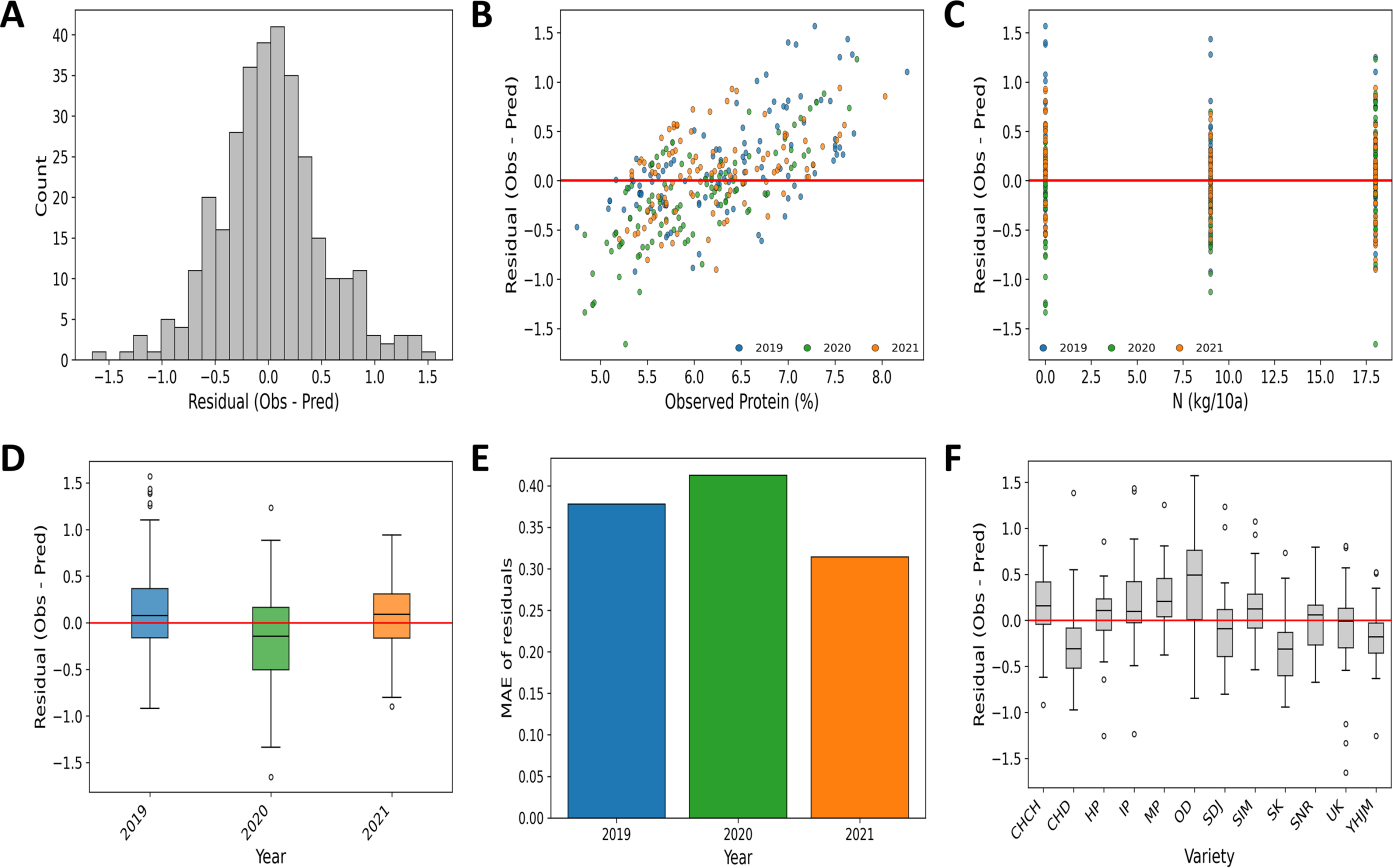
Residual analysis of rice grain protein content predictions obtained from the LASSO trained on replicate-mean datasets. **(A)** Histogram of residuals (observed − predicted). Residuals plotted against **(B)** observed rice grain protein content and **(C)** nitrogen application rate. Residual distributions across experimental years **(D)**, mean absolute error of residuals by year **(E)**, and residual distribution across cultivars **(F)**. Cultivar names are presented as abbreviations; full cultivar names and characteristics are provided in **Supplementary Table 2**.

To complement the aggregate performance metrics and evaluate the model reliability under specific conditions, residual diagnostics were conducted using the LASSO trained on the replicate-mean dataset under LOYO cross-validation. The distribution of the residuals was approximately symmetric and centered around zero, with no strong skewness and only limited extreme deviations (**Figure 8A**), indicating the absence of pronounced systematic overestimation or underestimation.

Residual behavior was further examined to assess dependent on protein magnitude and management intensity. Across the observed range of grain protein contents, residuals showed no nonlinear patterns (**Figure 8B**). However, residual dispersion increased modestly at higher protein levels, suggesting a degree of heteroscedasticity. Despite this, residuals remained centered around zero, indicating that prediction errors were not systematically biased toward specific protein range.

Similarly, residuals across nitrogen application rates showed no consistent directional bias (**Figure 8C**). Although variability increased slightly at higher nitrogen levels, the absence of systematic shifts suggests that the model adequately captured nitrogen effects without overemphasizing specific management regimes.

Residuals were further analyzed across experimental years to assess potential temporal bias. Residual distributions remained centered around zero for all years, with broadly comparable spread across 2019, 2020, and 2021 (**Figure 8D**). The mean absolute error of residuals showed moderate variation among years (approximately 0.38, 0.41, and 0.32 for 2019, 2020, and 2021, respectively; **Figure 8E**) indicating that predictive performance was relatively consistent despite inter-annual climatic variability.

Residuals stratified by cultivar showed distributions generally centered around zero (**Figure 8F**), suggesting no strong cultivar-specific bias. Although some cultivars exhibited wider dispersion, no consistent directional bias was observed.

**Figure 9:**
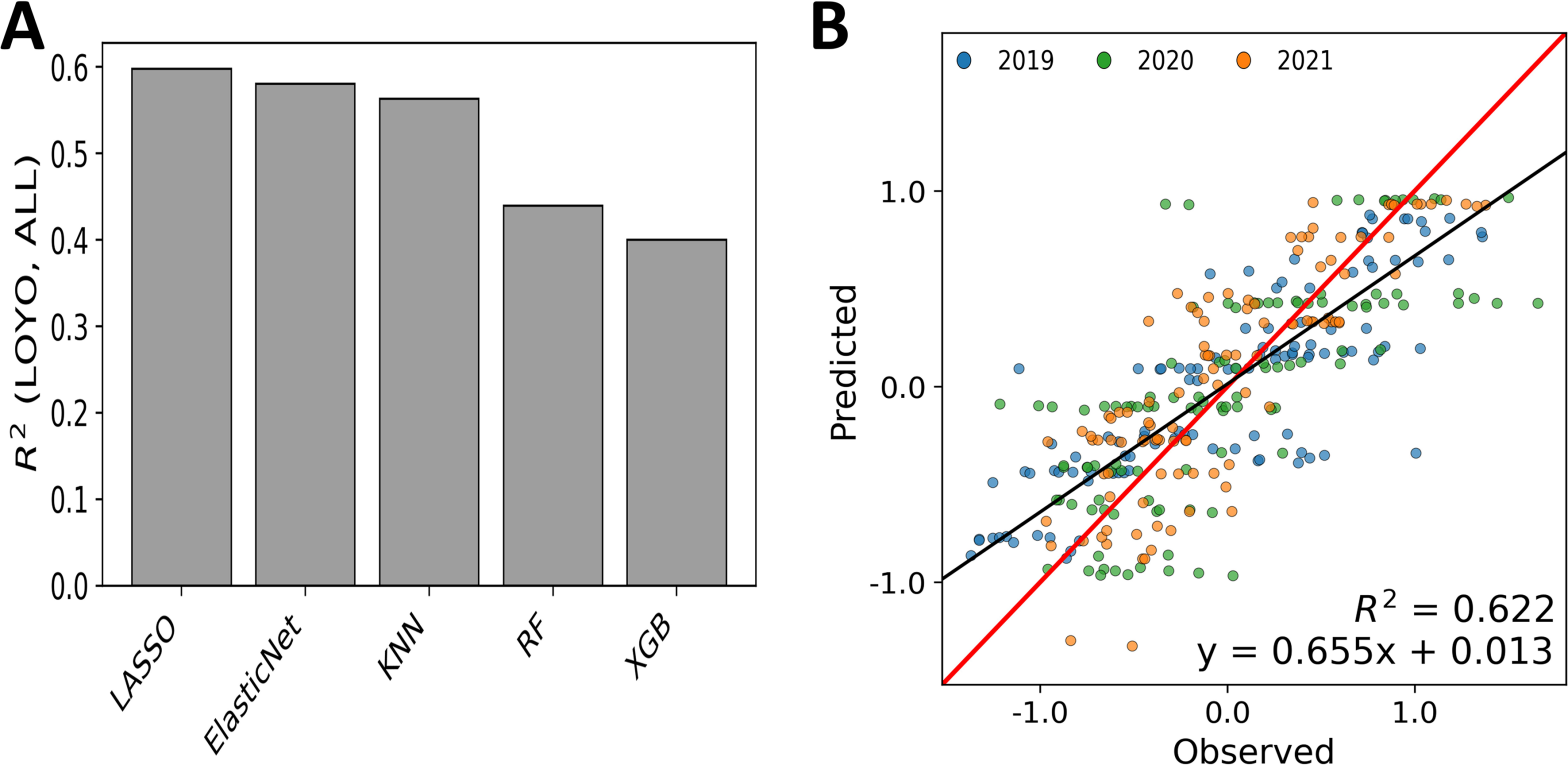
Prediction performance of rice grain protein content anomalies, defined as deviations from year–variety baselines, using replicate-mean datasets. **(A)** Model comparison in terms of R² under Leave-One-Year-Out (LOYO) cross-validation. **(B)** Observed-versus-predicted protein anomalies for the LASSO. LASSO, Least Absolute Shrinkage and Selection Operator; KNN, k-nearest neighbors; RF, Random Forest; XGB, Extreme Gradient Boosting.

To further evaluate model behavior beyond absolute prediction accuracy, anomaly-based analysis was conducted. Protein anomalies were defined as deviations from the year–variety-specific baseline, emphasizing relative variation within each year. Under this framework, all evaluated models retained meaningful predictive skills, with R^2^ values generally exceeding 0.45 (**Figure 9A**).

The observed-versus-predicted anomaly plots demonstrated a clear positive association, although deviations from the one-to-one line were evident (**Figure 9B**). This indicates that the models captured relative variation effectively, while exhibiting some underestimation at higher anomaly values.

Collectively, these results indicate that the modeling framework provides reasonable performance for both absolute protein level and relative anomaly patterns. This capability is particularly relevant for agronomic applications, where capturing relative differences among treatments within a given season is often more important than precise absolute predictions.

### 3.8 Evaluation of anomaly-based reconstruction

**Figure 10:**
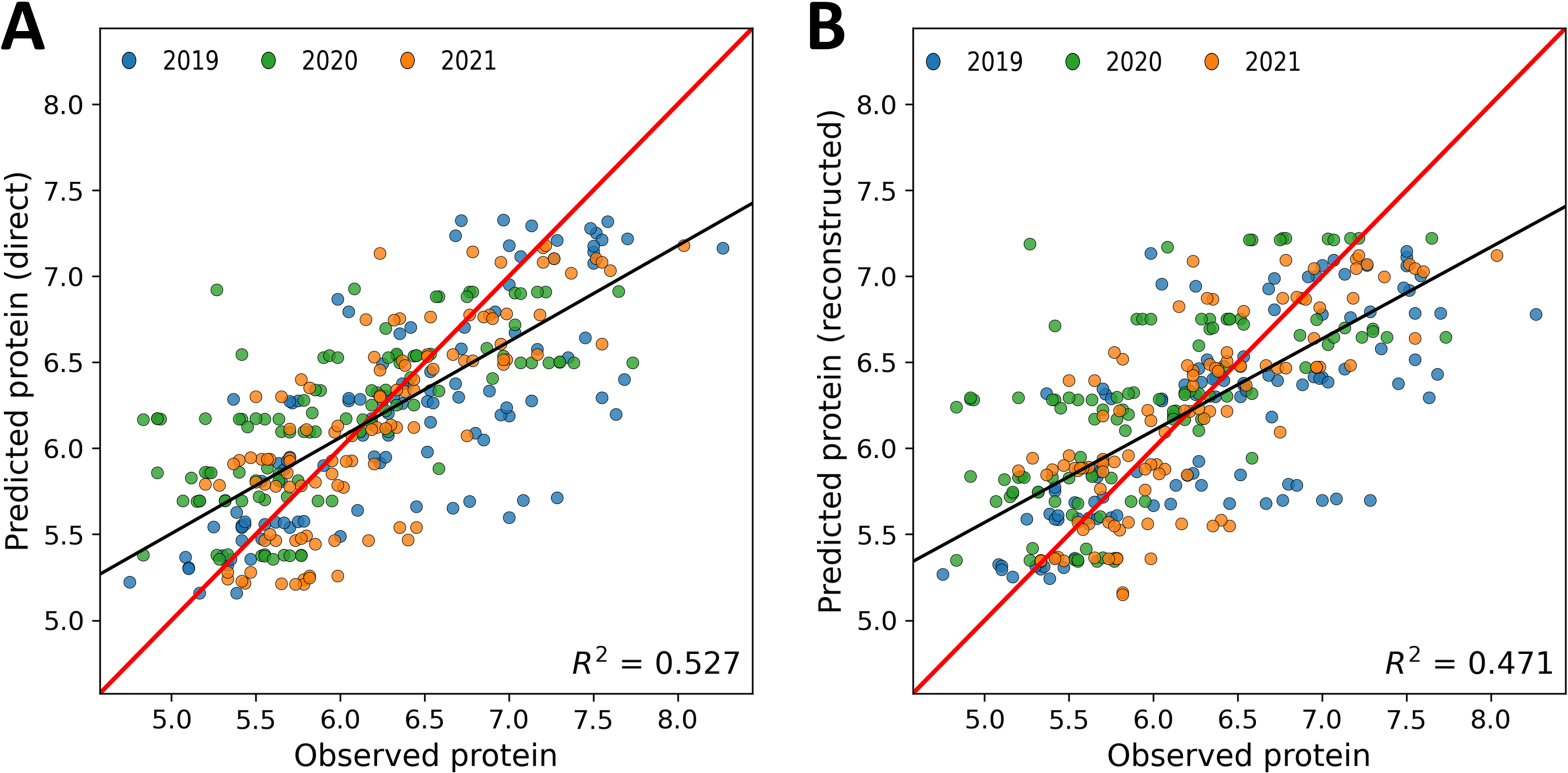
Comparison between direct protein prediction and anomaly-based reconstructed prediction under LOYO validation. **(A)** Observed versus predicted protein content for the direct prediction model. **(B)** Observed versus reconstructed protein content obtained by adding the predicted anomaly to the year-specific baseline protein level.

To evaluate the practical contribution of the anomaly-based framework, we compared the predictive performance of the direct protein prediction model with that of a reconstructed model obtained by adding the predicted anomaly to the year-specific baseline protein level. The reconstructed model did not improve the overall predictive performance relative to the direct model. Under LOYO validation, the direct model achieved an R² of approximately 0.53, whereas the reconstructed model showed a lower R² of approximately 0.47 (**Figure 10**).

These results indicate that anomaly-based reconstruction did not improve absolute prediction accuracy in this study when used for direct reconstruction of protein content. However, this finding clarifies the role of the anomaly-based framework. Rather than improving absolute prediction accuracy, anomaly analysis provides complementary interpretive value by isolating relative deviations from dominant year effects, thereby supporting the assessment of management-driven variability under heterogeneous field conditions.

## 4 Discussion

Multi-year field data revealed moderate to substantial variability in rice grain protein content driven by both environmental and management factors. This variability, widely reported in previous studies, reflects the sensitivity of protein accumulation to climatic conditions during grain-filling, particularly under open-field environments (Huang et al., 2019; Liu et al., 2020; Shimoyanagi et al., 2021). Such complexity underscores the challenge of predicting grain quality under realistic field conditions and highlights the limitations of evaluation approaches that ignore temporal structures.

Climatic differences among experimental years, including precipitation-dominant and high temperature conditions, provided a realistic basis for evaluating year-independent model generalization. Climatic variables were structured into non-overlapping time windows (0–20 and 21–40 days after heading), reducing multicollinearity and enabling stage-specific interpretation. Late grain-filling conditions contributed more strongly to prediction performance, consistent with their role in nitrogen remobilization and grain development, as supported by feature-importance analysis.

Despite this, overlapping protein distribution across years indicates that climatic effects were not expressed in isolation but interacted with management and cultivar factors. Nitrogen application and transplanting time likely modulated climatic impacts, consistent with previous findings showing climate-induced changes in nitrogen dynamics (Tantray et al., 2022; Jauregui et al., 2025). Feature-group comparisons further confirmed that management variables accounted for the majority of predictive performance, while climatic variables provided complementary explanatory power, supporting a management-driven framework with climatic adjustment rather than a purely climate-driven model.

Feature-importance analysis further highlighted the role of late-stage temperature and radiation, indicating that the model captured agronomically plausible relationships between environmental conditions and protein formation. These results, together with year-wise environmental differences, help explain why models validated under random or within-year schemes often fail to generalize across seasons.

A key contribution of this study is demonstrating that data structuring aligned with agronomic design is as critical as model selection. Aggregating replicate observations improved stability under LOYO validation, indicating that replicate means better capture treatment-level responses by reducing microscale variability (Priyatikanto et al., 2023; Khatibi and Ali, 2024).

Validation comparison further showed that random split consistently overestimated predictive performance relative to LOYO. Even when controlling for test-set size, the performance gap persisted, indicating that it primarily reflects year-wise distribution shifts rather than reduced training data. Thus, LOYO provides a conservative but more realistic estimate of model generalization under unseen growing conditions (Hansen and Jones, 2000; Hernandez-Ochoa et al., 2024; Proctor et al., 2025).

Compared with previous studies reporting R² values of 0.60–0.80 under conventional validation, the lower performance observed (R² ≈ 0.45–0.52) reflects the more stringent evaluation framework and provides a more realistic assessment of model applicability across heterogeneous years.

Residual and anomaly-based analyses further demonstrated generally stable model behavior across protein levels, nitrogen rates, cultivars, and years. The absence of systematic bias supports model reliability, while anomaly-based analysis confirmed the ability to capture relative deviations from year-specific baselines. Although anomaly-based reconstruction did not improve absolute accuracy, it provides valuable interpretive insight under nonstationary conditions, consistent with previous studies (Liu et al., 2025; Wei et al., 2025).

From a practical perspective, the ability to estimate relative protein responses is particularly relevant for precision nitrogen management, where relative differences within a season are often more actionable than absolute values. The proposed framework can support adaptive nitrogen strategies under varying climatic conditions, improving nitrogen use efficiency and decision-making.

The present study has several limitations. The use of a 1.6 mm sieve may slightly overestimate protein values compared with larger thresholds, although consistent processing minimizes its impact on relative comparisons. In addition, the study did not explicitly include physiological or soil variables, which have been shown to improve prediction accuracy (Togninalli et al., 2023; Zhang et al., 2023; Rodrigues et al., 2024).

The LOYO validation in this study was based on only three experimental years, and fold-wise performance may remain sensitive to individual year conditions. Therefore, further validation across diverse environments and longer time series is required to obtain more stable estimates of temporal generalization.

Despite these limitation, the proposed framework provides a potentially generalizable analytical approach for crop quality prediction under heterogeneous environments. This framework can be extended to other crops, traits, and management systems, although further validation across diverse conditions is required.

Future studies should incorporate additional explanatory variables, including physiological indicators (e.g., SPAD), remote sensing-derived vegetation indices (e.g., NDVI or EVI), and nitrogen dynamics during grain filling. Such integration would enable a more mechanistic understanding of protein accumulation and further enhance predictive performance.

## 5 Conclusion

This study evaluated the year-independent predictability of rice grain protein content using multi-year field experimental data and machine learning approaches. By integrating replicate aggregation with year-independent validation, the proposed framework provided a more realistic assessment of model generalization under contrasting climatic conditions.

The results demonstrate that data structuring aligned with agronomic experimental design is as critical as model selection for achieving robust prediction performance. In particular, replicate aggregation improved model stability by reducing plot-level variability, while year-independent validation prevented the overestimation of predictive accuracy commonly observed in conventional approaches.

From a practical perspective, the proposed framework enables reliable estimation of grain protein responses and relative trends within seasons, supporting adaptive nitrogen management under variable climatic conditions.

Overall, this study provides a generalizable framework for applying machine learning to field-based crop quality prediction, highlighting the importance of integrating agronomic knowledge with data-driven approaches to achieve robust and interpretable models.

## Data Availability

The original contributions presented in the study are included in the article/**Supplementary Material**. Further inquiries can be directed to the corresponding author.
