# Supplementary material for: Year-independent prediction of rice grain protein content using machine learning with agronomy-aligned multi-year field data
Source: Front Plant Sci. 2026 Jun 1;17:1818096. doi: 10.3389/fpls.2026.1818096 (PMC13265279; doi:10.3389/fpls.2026.1818096)
Supplement: Supplementary file 1 [file Table1.docx]

Supplementary Table S1 Hyperparameter setting and optimization strategy for machine learning models

| **Model** | **Hyperparameter** | **Final value** | **Search range** | **Optimization method** | **Notes** |
| --- | --- | --- | --- | --- | --- |
| Elastic Net (EN) | alpha | 0.03 | 0.001–1.0 (log scale) | Grid search | Regularization strength |
|  | l1_ratio | 0.5 | 0.1–0.9 | Grid search | Balance between L1 and L2 penalties |
|  | max_iter | 20000 | fixed | - | Convergence setting |
| LASSO | alpha | 0.01 | 0.001–1.0 (log scale) | Grid search | L1 regularization strength |
|  | max_iter | 20000 | fixed | - | Convergence setting |
| Random Forest (RF) | n_estimators | 800 | 100–1000 | Grid search | Number of trees |
|  | min_samples_leaf | 2 | 1–10 | Grid search | Minimum samples per leaf |
| XGBoost (XGB) | n_estimators | 1200 | 100–1500 | Grid search | Number of boosting rounds |
|  | max_depth | 4 | 3–10 | Grid search | Tree depth |
|  | learning_rate | 0.03 | 0.01–0.3 | Grid search | Learning rate |
|  | subsample | 0.9 | 0.5–1.0 | Grid search | Row sampling ratio |
|  | colsample_bytree | 0.9 | 0.5–1.0 | Grid search | Feature sampling ratio |
| k-nearest neighbors (KNN) | n_neighbors | 15 | 3–25 | Grid search | Number of neighbors |
|  | weights | distance | {uniform, distance} | Grid search | Weighting scheme |
