# Supplementary material for: Year-independent prediction of rice grain protein content using machine learning with agronomy-aligned multi-year field data
Source: Front Plant Sci. 2026 Jun 1;17:1818096. doi: 10.3389/fpls.2026.1818096 (PMC13265279; doi:10.3389/fpls.2026.1818096)
Supplement: Supplementary file 2 [file Table2.docx]

Supplementary Table S2 Descriptive statistics of rice grain protein content for the entire data set and stratified by year, variety, and nitrogen application rate.

| **Factor** | **Level** | **Mean** | **SD** | **Min** | **Max** |
| --- | --- | --- | --- | --- | --- |
| Overall | All | 6.19 | 0.78 | 3.1 | 8.65 |
| Year | 2019 | 6.36 | 0.83 | 3.65 | 8.65 |
|  | 2020 | 5.99 | 0.77 | 3.1 | 8.1 |
|  | 2021 | 6.22 | 0.68 | 5.1 | 8.2 |
| Nitrogen^*^  (kg·10a⁻¹, equivalent to kg·ha⁻¹) | 0 | 5.66 | 0.53 | 3.1 | 7.4 |
|  | 9 | 6.11 | 0.62 | 3.65 | 8.65 |
|  | 18 | 6.8 | 0.7 | 4.9 | 8.5 |
| Transplanting Time | Early | 5.84 | 0.64 | 4.7 | 7.9 |
|  | Late | 6.54 | 0.79 | 3.1 | 8.65 |
|  | Moderate | 6.2 | 0.72 | 3.65 | 8.1 |
| Variety^**^ | CHCH | 6.33 | 0.8 | 3.65 | 8 |
|  | CHD | 5.91 | 0.67 | 4.7 | 7.3 |
|  | HP | 6.24 | 0.83 | 4.4 | 8.1 |
|  | IP | 6.36 | 0.9 | 3.1 | 8.65 |
|  | MP | 6.46 | 0.78 | 5.25 | 7.85 |
|  | OD | 6.62 | 0.79 | 5.3 | 8.5 |
|  | SDJ | 6.08 | 0.8 | 5 | 8.1 |
|  | SIM | 6.29 | 0.61 | 5.15 | 7.7 |
|  | SK | 5.84 | 0.68 | 4.75 | 7.8 |
|  | SNR | 6.16 | 0.77 | 4.8 | 8.1 |
|  | UK | 6.07 | 0.66 | 4.75 | 7.6 |
|  | YHJM | 5.95 | 0.64 | 4.45 | 8.2 |

^*^Nitrogen application rate is expressed in kg·10a⁻¹, with equivalent values in kg·ha⁻¹ (1 kg·10a⁻¹ = 10 kg·ha⁻¹).

^**^Cultivar names are presented as abbreviations, with full names and characteristics described as follows: CHCH(Chucheong, Japonica, medium-to-late maturing cultivar); CHD(Chindeul, Japonica, medium-to-late maturing cultivar); HP(Hopum, Japonica, medium-to-late maturing cultivar); IP(Ilpum, Japonica, medium-to-late maturing cultivar); MP(Mipum, Japonica, medium-to-late maturing cultivar); OD(Odae, Japonica, early-maturing cultivar); SDJ(Sindongjin, Japonica, medium-to-late maturing cultivar); SIM(Seailmi, Japonica, medium-to-late maturing cultivar); SK(Samkwang, Japonica, medium-to-late maturing cultivar); SNR(Saenuri, Japonica, medium-to-late maturing cultivar); UK(Unkwang, Japonica, early-maturing cultivar); YHJM(Younhojinmi, Japonica, medium-to-late maturing cultivar)
