# Supplementary material for: Year-independent prediction of rice grain protein content using machine learning with agronomy-aligned multi-year field data
Source: Front Plant Sci. 2026 Jun 1;17:1818096. doi: 10.3389/fpls.2026.1818096 (PMC13265279; doi:10.3389/fpls.2026.1818096)
Supplement: Supplementary file 3 [file Table3.docx]

Supplementary Table S3 Comparison of model training time across machine learning algorithms. Values represent mean training time and standard deviation over repeated runs under identical computational conditions.

| Model | Mean training time (s)* | Standard deviation (s) | Relative ranking |
| --- | --- | --- | --- |
| Elastic Net | 0.12 | 0.01 | 1 |
| LASSO | 0.15 | 0.02 | 2 |
| KNN | 0.45 | 0.05 | 3 |
| RF | 1.85 | 0.20 | 4 |
| XGB | 3.10 | 0.35 | 5 |

* All models were trained and evaluated under identical computational conditions using a workstation equipped with an Intel Core i9-14900KF CPU (3.20 GHz), 128 GB RAM, and an NVIDIA GeForce RTX 5080 GPU, running Windows 11 Pro (64-bit).
