# Supplementary material for: Year-independent prediction of rice grain protein content using machine learning with agronomy-aligned multi-year field data
Source: Front Plant Sci. 2026 Jun 1;17:1818096. doi: 10.3389/fpls.2026.1818096 (PMC13265279; doi:10.3389/fpls.2026.1818096)
Supplement: Supplementary file 4 [file Table4.docx]

Supplementary Table S4 Comparison of prediction performance between raw replicate-level data and replicate-mean data using Leave-One-Year-Out validation.

| **Dataset** | **^*^ML model** | **R^2^** | **RMSE** | **MAE** |
| --- | --- | --- | --- | --- |
| Raw | ElasticNet | 0.4513 | 0.5746 | 0.4471 |
|  | LASSO | 0.4604 | 0.5698 | 0.4394 |
|  | RF | 0.2558 | 0.6692 | 0.5201 |
|  | XGB | 0.3335 | 0.6333 | 0.4914 |
|  | KNN | 0.2465 | 0.6734 | 0.5197 |
| Rep_mean | ElasticNet | 0.5376 | 0.4833 | 0.3730 |
|  | LASSO | 0.5485 | 0.4775 | 0.3625 |
|  | RF | 0.3605 | 0.5683 | 0.4410 |
|  | XGB | 0.4136 | 0.5442 | 0.4174 |
|  | KNN | 0.3829 | 0.5583 | 0.4286 |

^*^ML models are abbreviated as follows: LASSO, Least Absolute Shrinkage and Selection Operator; RF, Random Forest; XGB, Extreme Gradient Boosting; KNN, k-nearest neighbors.
